# Disrupted gut microbiome networks and unhealthy behaviors predict metabolic dysfunction in children and adolescents in the long term

**DOI:** 10.1016/j.isci.2026.114763

**Published:** 2026-01-24

**Authors:** Silvia Turroni, Kathrin Günther, Federica D’Amico, Toomas Veidebaum, Yiannis Kourides, Dénes Molnár, Lauren Lissner, Ronja Foraita, Monica Barone, Carlos Mora-Martínez, Yolanda Sanz, Arno Fraterman, Maike Wolters, Patrizia Brigidi, Marco Candela, Wolfgang Ahrens, Simone Rampelli

**Affiliations:** 1 Microbiome Science and Biotechnology Unit, Department of Pharmacy and Biotechnology, University of Bologna, 40126 Bologna, Italy; 2 Leibniz Institute for Prevention Research and Epidemiology - BIPS, 28359 Bremen, Germany; 3 Department of Chronic Diseases, National Institute for Health Development, 10617 Tallinn, Estonia; 4 Research and Education Institute of Child Health, 2051 Strovolos, Cyprus; 5 Department of Paediatrics, Clinical Center, University of Pécs, 7623 Pecs, Hungary; 6 School of Public Health and Community Medicine, Institute of Medicine, Sahlgrenska Academy, University of Gothenburg, 40530 Gothenburg, Sweden; 7 Human Microbiomics Unit, Department of Medical and Surgical Sciences, University of Bologna, 40138 Bologna, Italy; 8 Microbiome Innovation in Nutrition and Health Research Unit, Institute of Agrochemistry and Food Technology, Spanish National Research Council (IATA-CSIC), 46980 Valencia, Spain; 9 Medizinisches Versorgungszentrum Dr. Eberhard & Partner Dortmund, Laboratoriumsmedizin Dortmund, 44137 Dortmund, Germany

**Keywords:** genomics, transcriptomics, microbial genomics

## Abstract

We recently showed that the individual gut microbiome (GM) configuration in children and adolescents, together with long-term dietary habits, can predict the development of obesity. Here, we expanded our previous cohort to include 218 individuals and used 16S rRNA amplicon sequencing, shotgun metatranscriptomics, and a network approach to analyze fecal samples collected at a baseline survey and after a 4-year follow-up, investigating associations with health status, dietary intake, and other health-related behaviors. Our results showed that an unbalanced GM profile in children/adolescents, with few represented species, poor connectivity, and low transcriptional activity (especially in relation to molecular effectors that positively influence gut and immune health), combined with unhealthy behaviors (i.e., low-fiber diet and reduced physical activity), may favor the onset of obesity. This knowledge may pave the way for the development of adjunct GM-based precision intervention strategies aimed at rewiring microbial networks to promote long-term health.

## Highlights

• Disrupted gut microbiome networks and unhealthy behaviors may favor obesity
• Few, poorly connected taxa with low activity are risk factors for metabolic health
• A diverse and connected microbiome may protect against metabolic dysfunction
• Medium-to-high levels of healthy eating and physical activity provide protection

## Graphical abstract

**Figure:**
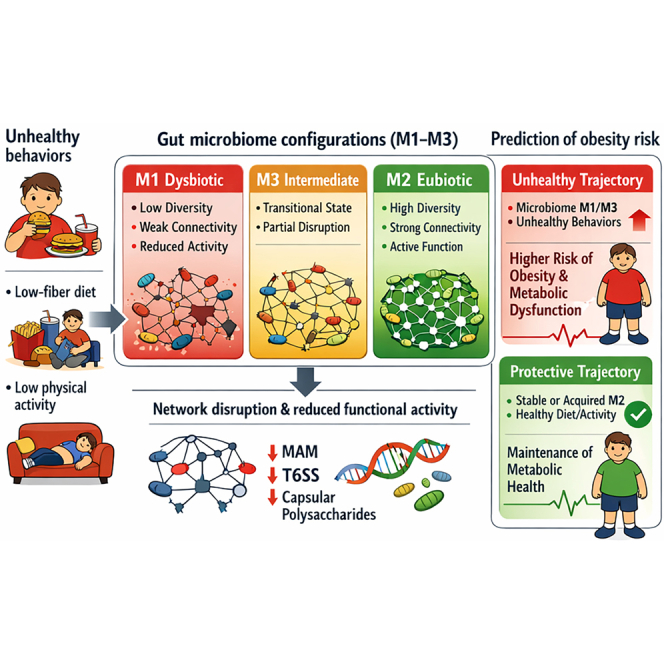

## Introduction

The gut microbiome (GM), i.e., the large ensemble of microorganisms, mainly bacteria, that inhabit the gastrointestinal tract, is undoubtedly recognized as a key player in overall health, influencing metabolism and the development and fine-tuning of the immune and central nervous systems.^1^ This process begins in the first hours after birth, when the GM starts to colonize the newborn’s gut, depending on multiple perinatal and postnatal exposures, including delivery mode, feeding regimen, antibiotic treatment, and the home environment (the so-called exposome).^2^ The composition and function of the GM then continue to fluctuate over the months until an adult-like structure is established.^3^^,^^4^^,^^5^^,^^6^^,^^7^ The importance of eubiotic GM development for long-term health is widely recognized in the literature, with early (and even transient) imbalances being potentially associated with metabolic, immunological, and neurological consequences later in life.^8^^,^^9^^,^^10^ However, researchers are still struggling to establish the correct developmental pathways and the time required for these to occur. As recently discussed, it is likely that the GM does not reach adult complexity in early childhood as was previously thought,^3^ but continues to evolve into adolescence and is more malleable to modification than in adulthood, providing additional opportunities for GM-based interventions to prevent deviance and promote health.^5^^,^^11^ Nevertheless, the composition and function of the GM in children and adolescents remain largely overlooked. Similarly, longitudinal studies are lacking, making it impossible to establish robust temporal associations between the GM, modifiable individual factors (e.g., diet and other lifestyle factors), and host health evolution.

In an attempt to address this knowledge gap, here we profiled the GM of 218 children/adolescents in a prospective study (from baseline to follow-up after 4 years), and investigated its correlations with dietary patterns, other lifestyle habits, and health parameters. Seventy of these individuals were from our previous study,^12^ while the remaining 148 were newly enrolled. The sample was derived from the IDEFICS (Identification and Prevention of Dietary- and Lifestyle-Induced Health Effects in Children and Infants) and I.Family (Investigating the determinants of food choice, lifestyle and health in European children, adolescents, and their parents) follow-up studies. In our previous work, we showed that the individual GM configuration in children/adolescents, together with long-term dietary habits, could serve as a predictive tool for obesity development. Here, we aimed to validate and extend the association between baseline GM configurations (alongside health, lifestyle behavior, and diet) and subsequent weight status. By integrating the 16S rRNA amplicon sequencing analysis with the additional fecal samples processed herein (for a total of 310 samples analyzed overall), we confirmed that the GM of children/adolescents can be stratified into a discrete number of clusters characterized by different levels of diversity and compositional structure, as well as different associations with diet, other lifestyle factors, and health, including obesity. Furthermore, we applied a network approach and shotgun metatranscriptomics to a sample subset and found that the most dysbiotic GM cluster (i.e., the cluster with the lowest diversity and the most unbalanced composition, associated with unhealthy behaviors) was also the most ecologically and functionally impaired. In particular, this cluster exhibited poorly connected species with low transcriptional activity, especially in relation to molecular effectors known to positively impact gut and immune health. The presence (and persistence over time) of such a cluster was associated with a higher likelihood of developing obesity.

## Results

### Gut microbiome structure in children/adolescents, and correlation with health, lifestyle behaviors, and diet

We previously identified large inter-individual variability in the GM structure of 70 children and adolescents (aged 4–14 years) sampled at two time points (T1 and T3) within a 4-year window.^12^ In particular, we found four GM configurations (C1 to C4) that were closely associated with human health. C1 and C2 were characterized by high diversity and an association with lower inflammation and a diet lower in carbohydrates, while C3 and C4 were associated with obesity development. To further explore the links between diet, behaviors, health, and GM, we extended our previous dataset to include a further 148 children/adolescents and 170 fecal samples, bringing the total number of individuals to 218 and the total number of fecal samples to 310 (218 at T1 and 92 at T3). This was paired with information on health status, dietary intake, and other health-related behaviors. Please, see Table 1 for the characteristics of the study cohort, and Tables S1 and S2 for the characteristics of participants who did or did not provide a fecal sample at T3. In addition to 16S rRNA amplicon sequencing, we used shotgun metatranscriptomics to achieve high-resolution taxonomic and functional profiling of the active GM fraction. The full cohort of 218 participants was stratified by measurement time and weight status as follows: i) normal weight at T1 and T3, *n* = 91; ii) overweight/obese at T1 and T3, *n* = 20; iii) underweight at T1 and T3, *n* = 18; iv) normal weight at T1 and excess weight gain at T3, *n* = 51; v) downward change in weight category at T3 compared to T1, *n* = 35; and vi) underweight at T1 and normal weight at T3, *n* = 3.

**Table 1:** Characteristics of the study cohort

	N	% or mean (range)
Sex, % (male/female)	116/102	53.2/46.8
Age in years at first examination, mean (range)	218	8.1 (4.1–11.1)

**Weight category at first examination, %**		

- Obese	15	6.9
- Overweight	28	12.8
- Normal weight	142	65.1
- Underweight	33	15.1

**Educational level of parents, %**		

- Low and medium	94	43.1
- High	124	56.9

**Figure 1:**
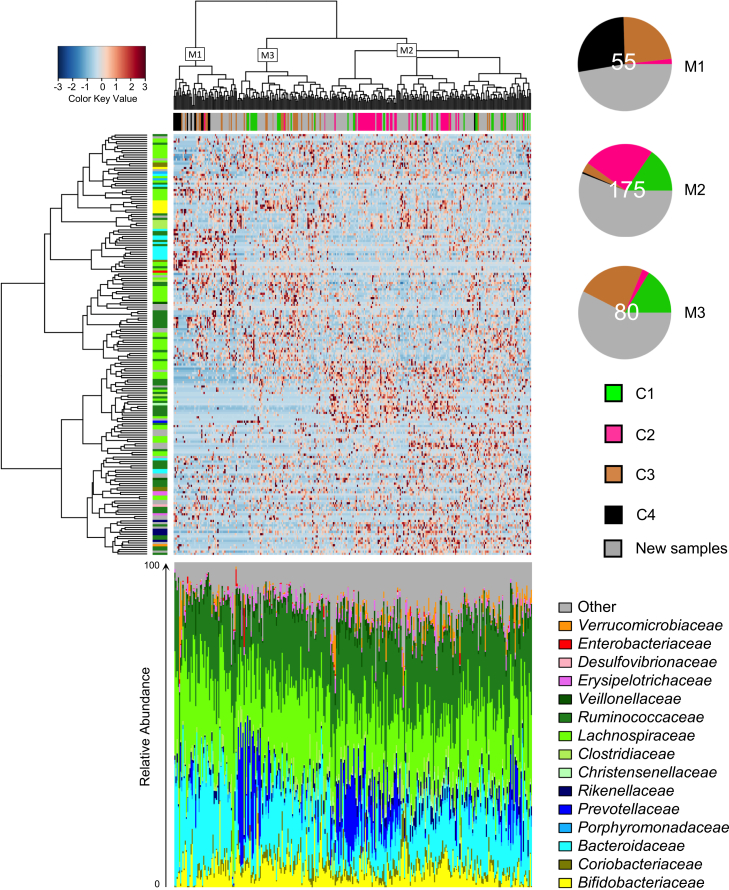
Variation in the gut microbiome of children/adolescents is summarized by three different configurations Top, Hierarchical Ward linkage clustering based on Spearman correlation coefficients of relative abundance of OTUs, filtered for OTU presence in at least 30% of individuals. Individuals are colored based on their gut microbiome configuration in our previous study (C1 to C4, Rampelli et al.,^12^). New samples are colored grey. The new gut microbiome configurations (M1 to M3) were defined based on the top tree and are highlighted with white squares. OTUs are color-coded according to their family assignment in the vertical tree. One hundred and ninety-one OTUs classified at the family level are visualized. On the right side of the heatmap, pie charts show the proportion and number of individuals for each of the new gut microbiome configurations. Bottom, Bar plots show the family-level gut microbiome profiles of the same samples present in the heatmap.

We generated approximately 3.8 million sequence reads from the V3-V4 regions of the 16S rRNA gene, with an average of 12,390 (±1,603 SD) high-quality reads per sample. Clustering analysis of operational taxonomic unit (OTU) relative abundances showed a clear separation between GM profiles, which partially overlapped with that obtained in our previous work (*p* = 0.0001, Fisher’s exact test).^12^ Indeed, when we integrated the previous results with the larger amount of data generated in the present study, we found that the previous four GM groups (C1 to C4) could be summarized into three new clusters (M1 to M3). Specifically, most of the previous C3/C4 configurations fell into the new M1 cluster, while most of the previous C1/C2 configurations fell into the new M2 cluster. The new M3 cluster occupied an intermediate position, containing numerous samples that had previously been assigned to the C1 and C3 configurations (see Figure 1; Table S3 for the correspondence between the previous C1-C4 clusters and the new M1-M3 clusters, as well as the demographic and anthropometric characteristics of the individuals at the sampling timepoints). Such subdivision into M1-M3 configurations appeared to be independent of stratification by time and weight status (Figure S1). However, when we compared the GM of individuals with normal weight at T1 who developed obesity at T3 with that of individuals with normal weight at T1 who remained normal weight at T3, we found that the former had a higher frequency of M1/M3 configurations than M2 (M1/M3 = 55% and M2 = 45%), while the opposite was true for the latter (M1/M3 = 39% and M2 = 61%) (*p* = 0.08, Fisher’s exact test).

**Figure 2:**
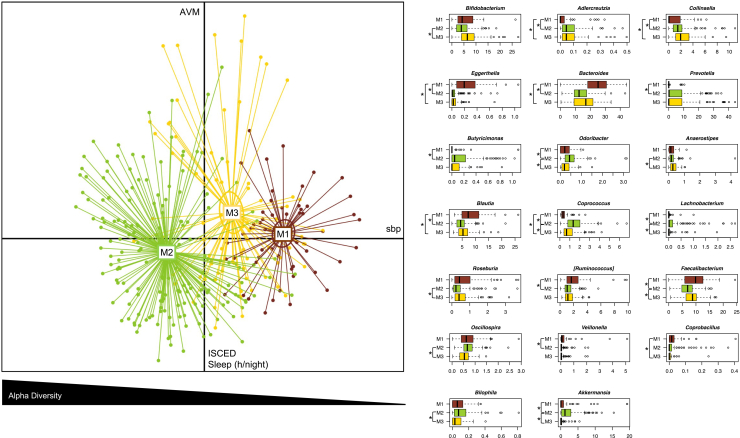
The three gut microbiome configurations of children/adolescents differ in taxonomic composition and are differentially associated with health and related behaviors Left, principal coordinates analysis (PCoA) plot (PCo1 and PCo3 axes used) showing three significantly different gut microbiome configurations of children/adolescents (M1 to M3, *p* < 0.001, permutation test with pseudo-*F* ratio), as defined by the unweighted UniFrac analysis of the whole cohort. The triangle below the PCoA indicates a decrease in alpha diversity (estimated using Faith’s phylogenetic diversity) from negative to positive values of the PCo1 axis, which is accompanied by a variation in systolic blood pressure (sbp). Variation in behavior assessment indices (hours of sleep per night – Sleep (h/night), International Standard Classification of Education of the parents – ISCED, and estimation of hours per day the child/adolescent usually spent on PC or watching television – AVM score) was associated with the PCo3 axis. See also Table 2. Right, Boxplots showing the relative abundance distribution of bacterial genera differentially represented between gut microbiome configurations (M1 to M3). ∗*p* < 0.05, Wilcoxon test. Boxplots represent the median (center line), the interquartile range (box, 25th–75th percentiles), and the whiskers extend to the most extreme values within 1.5× the interquartile range. Points beyond the whiskers represent outliers.

The UniFrac beta diversity and composition analyses allowed us to highlight the peculiarities of each GM configuration and their relationship with health and behavioral covariates (Figure 2). In particular, M1 was featured by a higher contribution of *Eggerthella*, *Bacteroides,* and *Blautia* and a lower contribution of *Adlercreutzia*, *Coprococcus*, and *Collinsella* compared to M2 and M3 (*p* < 0.05, Wilcoxon test). The M1 configuration also showed a higher relative abundance of *Coprobacillus* compared to M3, *Veillonella,* and *[Ruminococcus]* (family *Lachnospiraceae*) compared to M2, and a lower relative abundance of *Prevotella* and *Butyricimonas* compared to M2 (*p* < 0.05). Conversely, M2 showed higher proportions of *Odoribacter*, *Lachnobacterium*, *Coprococcus*, and *Akkermansia* compared to both M1 and M3 (*p* < 0.05). In addition, M2 showed a higher contribution of *Oscillospira* and *Bilophila* compared to M3, and a lower contribution of *Anaerostipes*, *Roseburia*, and *Bifidobacterium* compared to M3 (*p* < 0.05). Finally, both M1 and M3 showed a higher contribution of *Faecalibacterium* compared to M2 (*p* < 0.05). Correlations between health measures, health-related behaviors, and GM components were examined using quantile (median) regression tests and adjusted for sex, age, and country of origin. Median regression was chosen because it gives less weight to extreme values than linear regression, meaning that the results are less influenced by outliers. Significant associations between host covariates and major separations from the unweighted UniFrac analysis are shown in Table 2 and reported on the corresponding principal coordinates analysis (PCoA) axes in Figure 2. In particular, we found that a shift in GM toward positive values of the PCo1 axis was associated with systolic blood pressure. PCo1 was also negatively correlated with alpha diversity, i.e., higher values discriminated the M2 configuration, lower values M1, and intermediate values M3 (P = 1E-12, Kruskal-Wallis test with Faith’s diversity).

**Table 2:** Regression tests of associations between gut microbiome composition, clinical measurements, and behavioral metadata

Unweighted UniFrac PCoA for all individuals									
Parameter (n, number of samples used to compute the correlations)	PCo1			PCo2			PCo3		
	RC range	RC se	Pval	RC range	RC se	Pval	RC range	RC se	Pval
ISCED (education level score) (*n* = 302)	−0.02	−0.13	0.9	−0.14	−1.59	0.1	**−0.45**	**−1.59**	**0.03**
HDL (*n* = 259)	−0.15	−1.10	0.3	**−0.28**	**−3.06**	**0.002**	−0.10	−0.55	0.6
AVM (time spent with audiovisual media - PC and TV) score (*n* = 204)	−0.14	−0.78	0.4	−0.07	−1.19	0.2	**0.48**	**2.34**	**0.02**
Sleep (h/night) (*n* = 290)	−0.23	−1.32	0.2	0.18	1.31	0.2	**−0.42**	**−2.24**	**0.03**
Systolic blood pressure (*n* = 306)	**0.31**	**2.43**	**0.01**	0.10	0.94	0.4	0.15	0.65	0.5

HDL, high-density lipoprotein cholesterol; ISCED, International Standard Classification of Education of the parents.

Quantile (median) regression tests of associations between metadata measurements and gut microbiome composition as measured by unweighted UniFrac PCoA for all individuals. Tests were adjusted for sex, age, and country of origin. Column headings are: RC range, regression coefficients scaled to the full variation along each PCoA axis, thus indicating direction and magnitude of the association; RC se, regression coefficients scaled to one standard error; and Pval, quantile regression *p* value. The numbers in bold denote statistical significance.

**Figure 3:**
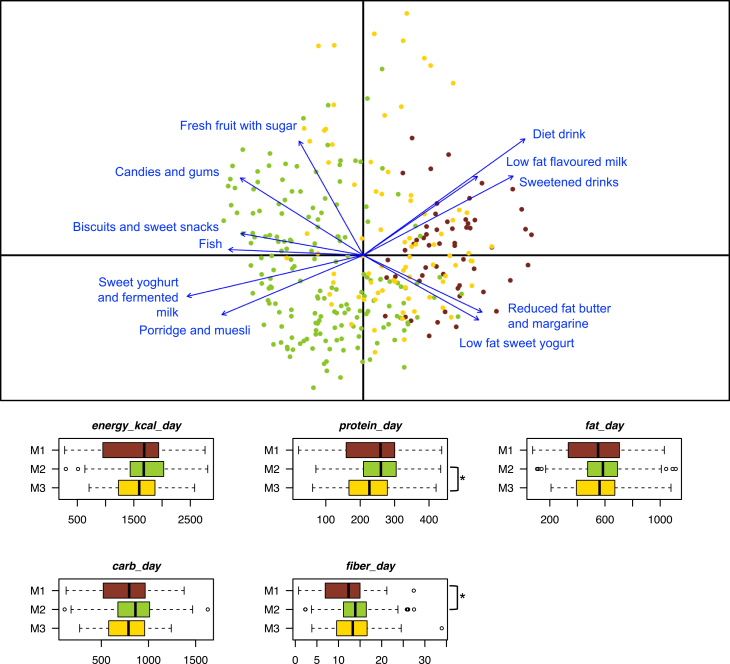
The three gut microbiome configurations of children/adolescents are differentially associated with food consumption (Top) PCoA based on unweighted UniFrac distances as in Figure 2, with the superimposition of the biplot of average food coordinates weighted by frequency of consumption per sample to identify the foods contributing to the ordination space (blue arrows). Only food categories that showed a significant correlation with sample segregation (*p* < 0.001, permutational correlation test) are shown. Samples are colored according to gut microbiome configuration (M1 to M3) as in Figure 2 bottom) Summary of macronutrient intake expressed as percentage of kilocalories consumed per day, fiber intake expressed as grams of fiber consumed per 1,000 kilocalories, and energy intake expressed as kilocalories per day. Data are shown for each of the three gut microbiome configurations. ∗*p* < 0.05, Wilcoxon test. Boxplots represent the median (center line), the interquartile range (box, 25th–75th percentiles), and the whiskers extend to the most extreme values within 1.5× the interquartile range. Points beyond the whiskers represent outliers.

To investigate which foods contributed most to the GM ordination in the three M1-M3 configurations (*p* < 0.001, permutational correlation test), food frequency questionnaire (FFQ) data were superimposed on the unweighted UniFrac PCoA plot of Figure 2 (Figure 3A). The M2 configuration was found to be associated with greater consumption of fruit, sweets, biscuits, snacks, sweet yogurt, fermented milk, porridge, muesli, and fish. Conversely, the M1/M3 configurations were associated with the consumption of reduced-fat butter and margarine, low-fat sweet yogurt, low-fat flavored milk, diet drinks, and sweetened drinks. In terms of macronutrients, individuals in the M2 configuration consumed more fiber and protein than those in the M1 and M3 configurations, respectively (*p* < 0.05, Wilcoxon test) (Figure 3B).

Taken together, these results confirm our previous findings (Rampelli et al. 2018) that certain GM configurations (i.e., M2), with higher alpha diversity and higher relative abundance of specific taxa, including *Akkermansia*, *Coprococcus*, *Prevotella*, *Odoribacter*, and *Lachnobacterium* (as the previous C1 and C2 configurations), are associated with favorable health-related behaviors, including, presumably, a healthier diet (particularly high in fiber), despite the consumption of sweets and snacks.

### Evidence of network disruption in gut microbiome configurations associated with unhealthy behaviors

**Figure 4:**
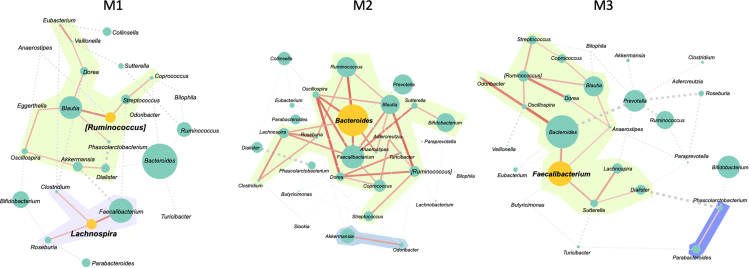
Disruption of network structure in gut microbiome configurations in children/adolescents is associated with unfavorable health-related behaviors Network plots corresponding to the three gut microbiome configurations of children/adolescents (M1 to M3), where disc size indicates the relative abundance of a given genus for each configuration, dashed gray lines represent significant negative correlations between taxa (*p* < 0.05), and red lines represent significant positive correlations (*p* < 0.05). The thickness and color intensity of the lines are proportional to the strength of the correlation. The subnetworks of bacteria with positive correlations are the modules and are represented by a green background and shades of purple. The discs are orange for keystone taxa and green for all other genera.

We then used network analysis to assess the GM structure in terms of positive or negative correlations, implying cooperation or competition between GM components (Figure 4). Different modules, i.e., subnetworks of bacteria with positive correlations, were detected. One central module contained generally dominant GM taxa, such as *Bacteroides*, *Faecalibacterium*, *Blautia*, *Bifidobacterium*, *Ruminococcus* (family *Ruminococcaceae*), *[Ruminococcus]*, *Dorea*, *Lachnospira*, *Oscillospira*, *Sutterella*, *Clostridium*, *Turicibacter*, *Anaerostipes*, *Adlercreutzia*, *Streptococcus*, and *Roseburia*. Such a module remained unchanged in the M2 configuration, but underwent derangement with the loss of some components in M1 and M3. In particular, the same module in M1 is split into two parts: the first contains *Blautia*, *Ruminococcus*, *[Ruminococcus]*, *Oscillospira*, *Coprococcus*, *Streptococcus,* and some bacteria that were previously only accessory, such as *Dialister* and *Eggerthella*; and the second contains *Faecalibacterium*, *Clostridium*, *Lachnospira*, and *Roseburia*. M3 showed an intermediate configuration between M1 and M2, with the loss of *Turicibacter*, *Clostridium*, *Adlercreutzia*, and *Roseburia*, and the entry of *Odoribacter* and *Dialister*. Topological data analysis revealed that *[Ruminococcus]* and *Lachnospira* in M1, *Bacteroides* in M2 and *Faecalibacterium* in M3 were keystone taxa, as they had the highest combination of closeness centrality (0.35, 0.37, 0.54 and 0.40, respectively), betweenness centrality (0.14, 0.11, 0.18 and 0.17) and degree (3, 3, 9 and 4), with an average relative abundance >1%. The variations in keystone taxa were the result of the gradual disintegration of the network structure, especially the main module, from M2 to M1 through M3. Indeed, the topological centrality values of the keystone taxa in M1 were much lower than those of the keystone taxon in M2. The same was true when comparing M3 with M2, albeit with less pronounced differences. This supports the hypothesis of the loss of GM components and cooperative relationships between microorganisms, rather than the gain of new relevant microorganisms in the network and the emergence of new equilibria, shifting from M2 to M3 and M1.

### Differences in the transcriptionally active fraction of gut microbiome configurations

**Figure 5:**
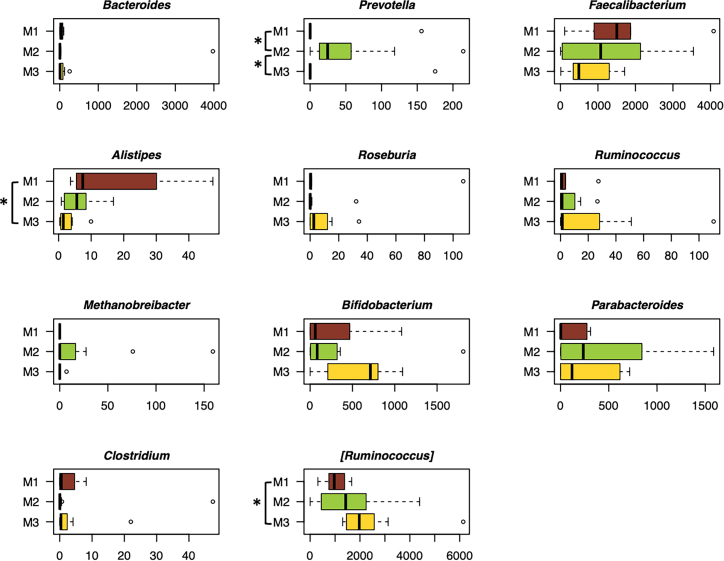
The three gut microbiome configurations in children/adolescents differ in transcriptionally active bacteria Boxplots show the distribution of transcript abundance for the 11 most representative genera found in the RNAseq analysis. The normalized abundance is expressed in coverage per million reads (CoPM), which is derived from the sum of the coverage of the specific contigs reconstructed from the RNAseq data assigned to the same genus in each sample, normalized to the total number of reads obtained from the sequencing. ∗*p* < 0.05, Wilcoxon test. Boxplots represent the median (center line), the interquartile range (box, 25th–75th percentiles), and the whiskers extend to the most extreme values within 1.5× the interquartile range. Points beyond the whiskers represent outliers.

To identify the transcriptionally active fraction of the GM, a subset of 25 samples (6 belonging to the M1 configuration, 12 to M2, and 7 to M3) were subjected to shotgun metatranscriptomics, yielding approximately 50 Gb of paired-end reads and 16,257 ± 1,268 (mean ± SEM) transcripts per sample. The metatranscriptomic dataset was dominated by 11 bacterial genera: *Bacteroides*, *Prevotella*, *Faecalibacterium*, *Alistipes*, *Roseburia*, *Ruminococcus*, *Methanobrevibacter*, *Bifidobacterium*, *Parabacteroides*, *Clostridium*, and *[Ruminococcus]*. Transcripts assigned to these genera, together with other contigs, for which no genus-level assignment could be obtained but which belonged to *Bacteroidaceae*, *Lachnospiraceae*, *Oscillospiraceae*, and Eubacteriales, accounted for around 76% of the total variability of the assigned sequences. These transcripts were distributed differently among the three configurations (M1-M3) (Figure 5). In particular, M2 was enriched in *Prevotella* transcripts compared to M1 and M3 (*p* ≤ 0.04, Wilcoxon test), which coincided with the 16S rRNA amplicon sequencing analysis. M1 was enriched in *Alistipes* transcripts and depleted in *[Ruminococcus]* transcripts compared to M3 (*p* = 0.02).

**Figure 6:**
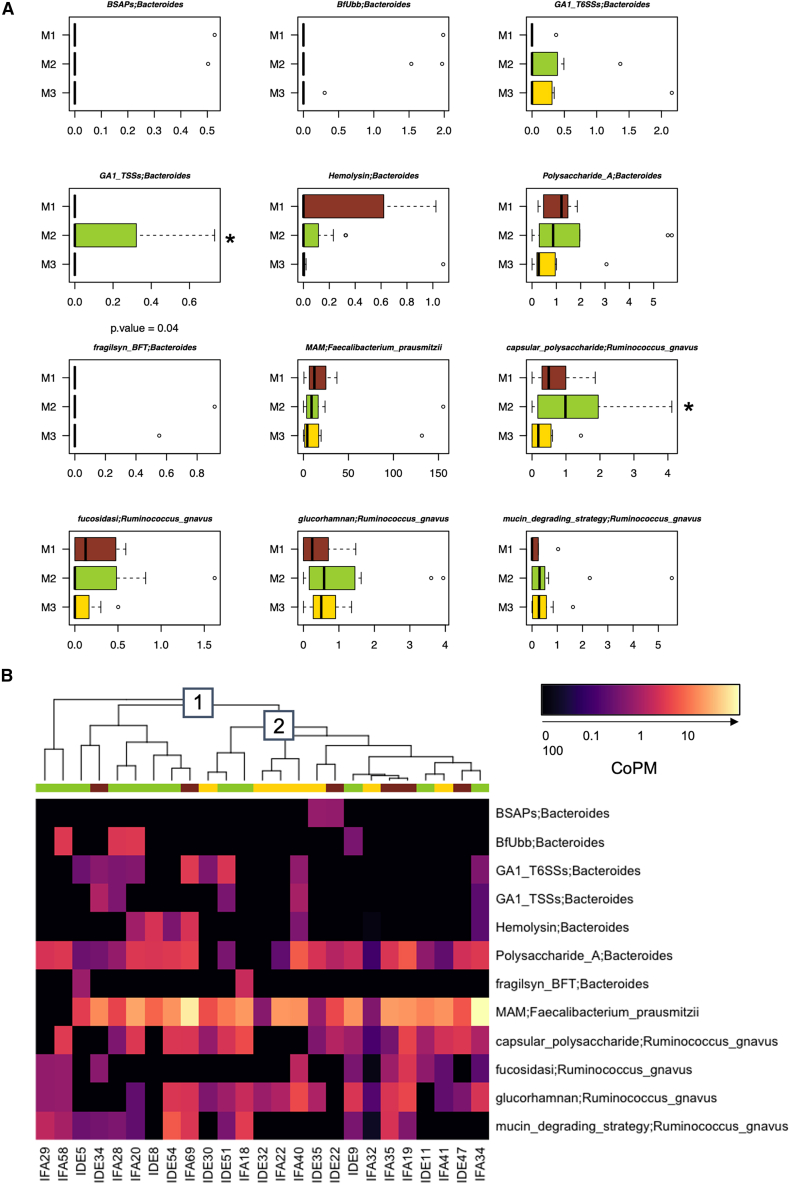
Transcript abundance for specific functions of keystone taxa differentiating the three gut microbiome configurations in children/adolescents (A) Boxplots show the abundance distribution of selected transcripts in coverage per million reads (CoPM), for the three different gut microbiome configurations (M1 to M3). ∗*p* < 0.05, Wilcoxon test. (B) Hierarchical Ward linkage clustering based on Spearman correlation coefficients of transcript abundance, expressed in CoPM. Two different transcript profiles (1,2) were defined based on the top tree and are highlighted with white squares. Boxplots represent the median (center line), the interquartile range (box, 25th–75th percentiles), and the whiskers extend to the most extreme values within 1.5× the interquartile range. Points beyond the whiskers represent outliers. See supplemental information, for the IDs of the genes investigated.

We then performed a detailed analysis of the transcriptomes of the 4 keystone taxa in our cohort (*[Ruminococcus]*, *Lachnospira*, *Bacteroides*, and *Faecalibacterium*), to determine whether their significance within the GM structure also implied functional relevance in their interaction with the human host. In particular, we focused on transcripts for microbial effectors that are already known in the literature to have a positive or negative impact on host health. Such a list included transcripts involved in the synthesis of: (i) the microbial anti-inflammatory molecule (MAM) for *Faecalibacterium prausnitzii*^13^; (ii) the inflammatory polysaccharide glucorhamnan,^14^ the ruminococcins A and C1-C5 (which play a role in colonization and persistence in the human gut),^15^^,^^16^ the capsular polysaccharide that promotes a tolerogenic immune response,^17^ the enzyme fucosidase for the degradation of L-fucose in the intestinal mucosa and other enzymes involved in mucin degradation^18^^,^^19^ for *Ruminococcus gnavus*; (iii) the lasso peptide with antimicrobial activity for *Lachnospira*^20^; and (iv) the Bacteroidales-secreted antimicrobial proteins BSAPs, the eukaryotic-like ubiquitin protein BfUbb (providing a competitive advantage in the gut via intraspecies antagonism), the type VI secretion systems (T6SSs) that release antimicrobial toxins, the toxins fragilysin and hemolysin, and the immunomodulatory polysaccharide A^21^^,^^22^ for *Bacteroides fragilis* and other *Bacteroides* species. See supplemental information for the IDs and sequences of the genes investigated. The transcripts for the above molecules were unevenly distributed among the three configurations (M1-M3), with those for *R*. *gnavus* capsular polysaccharide and *Bacteroides* T6SSs being overrepresented in M2 compared to M1 and M3 (*p* = 0.04) (Figure 6A). Clustering analysis of these transcripts revealed two distinct sample groups (Figure 6B). The first group, containing most of the M2 samples, presented almost all *Bacteroides* transcripts (except those for fragilysin and BSAPs), *R*. *gnavus* transcripts for the capsular polysaccharide, glucorhamnan, and the mucin degradation strategy, and the *F*. *prausnitzii* transcript for MAM. The latter group, containing mainly M1 and M3 samples, showed an overall depletion of *Bacteroides* transcripts (except those for polysaccharide A) and *R*. *gnavus* transcripts involved in the mucin degradation strategy (except those for fucosidase), while the other *R*. *gnavus* and *F*. *prausnitzii* transcripts were present at levels comparable to those in the first group. Notably, transcripts reported in the above list but not shown in Figure 6 were not detected in our metatranscriptomes. Taken together, these results suggest that keystone taxa of different GM configurations have the potential to differentially affect host health, particularly in terms of modulating immune and inflammatory responses and conferring competitive advantages in complex microbial communities.

### Correlating longitudinal changes in gut microbiome configurations and lifestyle behaviors with the development of obesity

Finally, our focus was on children and adolescents for whom fecal samples were available at both timepoints (T1 and T3), particularly those who were of normal weight at T1, either remaining so or gaining excessive weight at T3. We aimed to assess whether the prevention or onset of obesity was associated with a specific combination of GM configuration and lifestyle behaviors (i.e., dietary patterns and physical activity). In brief, for diet and physical activity, individuals were stratified into groups with high, medium, or low Healthy Diet Score (HDS) and moderate-to-vigorous physical activity (MVPA) values, respectively (see STAR Methods for details); for GM, persistence of M2 or conversion from M1 or M3 to M2 was considered. Children/adolescents who exhibited a stable M2 configuration over time, or who acquired this configuration at T3 (starting from M1 or M3), and who had a medium-to-high HDS or MVPA value at T1, did not develop obesity (*n* = 71, *p* = 0.009, Fisher’s exact test). This longitudinal analysis also revealed a prospective association: individuals with the M2 configuration and medium-to-high HDS or MVPA values at T1 were significantly less likely to become obese, highlighting the predictive value of this combined profile (*n* = 142, *p* = 0.005).

## Discussion

Here, we validated and extended our previous findings regarding the relationship between GM, diet, and other lifestyle behaviors, and health outcomes in children/adolescents.^12^ By expanding our study cohort to 218 individuals, a large proportion of whom were sampled at two time points (at a baseline survey and 4 years later), we confirmed that the GM of children/adolescents can be stratified into a discrete number of significantly different compositional clusters, namely M1 to M3. These clusters were distinct in terms of diversity, compositional structure, and association with dietary patterns and other lifestyle behaviors, and health parameters. In particular, M1 was the most dysbiotic GM cluster, featured by the lowest diversity, higher proportions of *Bacteroides*, *Eggerthella,* and Blautia, and lower proportions of *Coprococcus*, *Collinsella*, and *Prevotella*, among others. As expected, M1 was associated with a presumably less healthy diet, including the consumption of sweetened drinks, diet drinks, and sweetened milk products. Notably, this configuration was shared by most of the individuals with normal weight who went on to develop obesity, potentially representing an early predisposing factor for metabolic disease. Conversely, M2 showed a eubiotic, highly diverse profile, mainly discriminated by higher relative abundances of *Akkermansia*, *Coprococcus*, *Prevotella*, *Odoribacter*, and *Lachnobacterium*, and lower amounts of *Roseburia*, *Anaerostipes*, and *Bifidobacterium*. As expected, M2 was associated with a healthier overall diet, including higher fiber intake, and maintenance of a normal weight throughout the study period. However, M2 was also associated with consuming sweets and snacks. While this suggests that overall dietary patterns (particularly fiber intake) may be more relevant than individual food items,^23^ this certainly deserves further investigation. Finally, M3 shared some features with both M1 and M2, probably representing a transitional state that may more easily be steered toward a favorable profile. Most of the compositional signatures described above are consistent with the available literature,^24^ including our previous study.^12^ In particular, *Bacteroides*, *Eggerthella*, and *Blautia* have previously been identified as dominant in dysbiotic, low-diversity, pro-inflammatory GM configurations in children/adolescents associated with an unhealthy diet, inflammation, and the onset of obesity (i.e., the previous C3/C4 clusters of Rampelli et al.,^12^), while *Prevotella*, *Coprococcus*, *Collinsella*, *Odoribacter*, and *Lachnobacterium* were overrepresented in eubiotic, high-diversity GM configurations (i.e., the previous C1/C2 clusters of Rampelli et al.,^12^). These GM features are most likely driven by diet, such as the M2-related overabundance of *Prevotella* and *Coprococcus*, which are known to ferment fiber and produce short-chain fatty acids, key players in maintaining metabolic and immunological homeostasis.^25^ On the other hand, the M1-associated *Bacteroides* genus is known to include bile-tolerant microbes capable of promoting lipid digestion and absorption and fermenting proteins with the production of branched-chain fatty acids,^26^ which are generally linked to insulin resistance, obesity, and inflammation.^1^ No less importantly, *Bacteroides* spp. can degrade mucus, which helps them to settle in the gut; however, excessive mucus degradation may compromise the gut barrier,^27^^,^^28^ potentially supporting the association with the development of barrier-related disorders such as obesity. Regarding the higher proportion of *Akkermansia* in the healthiest GM cluster (i.e., M2), this contrasts with our previous work, in which it was associated with obesogenic dysbiotic GM profiles (i.e., C3/C4 clusters).^12^ However, this finding is consistent with the results of almost all available studies in children/adolescents^24^ as well as adults,^29^ in which *Akkermansia* is a hallmark of metabolic health.^30^

In an attempt to delve deeper into the GM findings at the community and functional levels, we used a network approach to assess ecological stability in terms of positive and negative relationships (i.e., cooperation and competition), and shotgun metatranscriptomics to gain functional insights. Network analysis revealed that the eubiotic, highly diverse GM cluster (M2) was also ecologically healthier, as it conserved a central community module comprising generally dominant GM taxa that were highly interactive with each other. In contrast, the GM network of the M1 configuration showed signs of destructuring, with a loss of components and cooperative connectivity, potentially suggesting less resilience and more vulnerability to ecological disturbance.^31^^,^^32^ Again, M3 occupied an intermediate position, potentially representing a configuration that was less compromised and could recover more easily toward a state of equilibrium. The keystone taxa were *[Ruminococcus]* and *Lachnospira* in M1, *Bacteroides* in M2, and *Faecalibacterium* in M3. The identification of *[Ruminococcus]* as a crucial node in the most dysbiotic configuration is not surprising, as it is a mucus-degrading taxon that has been consistently suggested to contribute to inflammatory states, including obesity.^33^ Conversely, the identification of *Bacteroides* as a keystone taxon in the healthiest configuration contrasts with its greater relative abundance in M1. This most likely indicates the establishment of different microbial community interactomes depending on the context, with different cross-feeding networks and thus different behavior within the community and in interaction with the host. Interestingly, the above taxa were found to exhibit differential transcriptional activity within GM configurations, particularly with regard to effectors known to influence host health. Specifically, M2 was distinguished by transcripts for *R*. *gnavus* capsular polysaccharide and *Bacteroides* GA1 T6SSs. The protective capsule of certain *R*. *gnavus* strains has been shown to elicit a tolerogenic immune response, imparting commensal behavior and promoting a symbiotic relationship with the host.^17^ Conversely, its absence has been shown to induce robust pro-inflammatory responses in both *in vitro* and *in vivo* mouse experiments. Concerning GA1 T6SSs, their loci are widespread across *Bacteroides* species due to their presence on integrative and conjugative elements.^34^ Although target cells have yet to be identified, it is plausible that, such as GA3 T6SSs, GA1 T6SSs act as contact-dependent antagonistic systems that increase the fitness of *Bacteroides* carriers, likely also enabling nutrient acquisition and conferring protection against environmental stressors.^35^ It is therefore tempting to speculate that the M2 configuration is also the most eubiotic from a transcriptional point of view, in terms of ecological advantages (including niche colonization) of some of its commensals and maintenance of gut homeostasis and symbiosis (by dampening inflammatory responses).

The study has the following merits: i) it investigated the GM of children/adolescents, an age group that is generally neglected in microbiome research despite its recognized importance for long-term health^11^; ii) it used a network approach, which is still underutilized in microbiome research,^36^ despite its ability to reveal GM connections and identify module gains and losses, providing insights into community ecology and behavior; and iii) it used metatranscriptomics to gain functional insights into transcriptionally active GM taxa and molecular effectors.

In conclusion, our study highlighted the importance of the individual GM configuration, in terms of diversity, compositional and functional structure, and community network, as a protective or risk factor for metabolic health in children/adolescents. In particular, an unbalanced GM profile, with few represented and poorly connected species and low transcriptional activity (especially in relation to tolerogenic immune pathways and gut fitness benefits), combined with unhealthy behaviors, may favor the onset of obesity. Conversely, maintaining or acquiring an eubiotic GM profile over time, characterized by higher diversity, network connectivity, and functional potential, alongside medium-to-high levels of healthy eating and physical activity, may protect against metabolic dysfunction. Strikingly, when examined prospectively, this combination (eubiotic GM profile and medium-to-high levels of healthy behaviors) showed predictive potential for the prevention of obesity during the study period. Future studies in larger cohorts, with more frequent longitudinal sampling and other omics (especially metabolomics), are needed to enable high-resolution temporal mapping of GM and its influence on human health. This knowledge will be instrumental in developing adjunct precision intervention strategies aimed at restoring GM diversity, rewiring microbial networks, and enhancing healthy behaviors to promote long-term health.

### Limitations of the study

The major limitations of the study include: i) the small sample size of some groups stratified by time and weight status (particularly those who were underweight at T1 and returned to normal weight at T3), although this was not a specific objective of our study; ii) the limited number of samples that could be subjected to shotgun metatranscriptomics; and iii) the lack of metabolomic/proteomic profiling to validate transcriptional information. Furthermore, despite the prospective cohort design, the GM analyses were primarily cross-sectional, focusing on the associations between GM configurations (alongside health, lifestyle behavior, and diet) at baseline (T1) and subsequent weight status. This was mainly due to: i) the reliance on our previous article,^12^ as the aim of the present study was to validate the previously identified cross-sectional associations; and ii) the failure to collect fecal samples from many participants at T3, which is common in children/adolescent studies, but limited the ability to conduct longitudinal assessments. Nevertheless, the 92 fecal samples collected at T3 were analyzed longitudinally to investigate overall GM cluster stability and its association with the likelihood of developing obesity.

## Resource availability

### Lead contact

Further information and requests for resources and reagents should be directed to and will be fulfilled by the lead contact, Simone Rampelli (simone.rampelli@unibo.it).

### Materials availability

This study did not generate new unique reagents.

### Data and code availability

• **Data:** sequencing reads were deposited as raw data along with available metadata in the ENA Archive (BioProject ID: PRJEB102780 for both 16S rRNA amplicon sequencing and shotgun metatranscriptomics). All relevant data are available from the authors. Sequencing reads from the first study (Rampelli et al. 2018) were available on MGRAST (https://www.mg-rast.org/mgmain.html?mgpage=project&project=mgp84098). Upon reasonable request, the lead contact can provide the data for other analyses.
• **Code:** No custom code was generated in this study.
• **Other items:** This study did not generate new unique reagents, materials, or resources.

## Acknowledgments

This study has been funded by the FP7-EU MyNewGut grant agreement No. 613979, and the “Severo Ochoa” grant of 10.13039/501100001665National Agency for Research (10.13039/501100011033AEI) - 10.13039/501100004837Spanish Ministry of Science and Innovation (Ref. CEX2021-001189-S).

## Author contributions

P.B., W.A., and S.R. designed the project. T.V., K.Y., D.M., L.L., R.F., C.M.M., and A.F. collected the samples. F.D.A. and M.B. performed the sequencing. S.R. ran the bioinformatics and biostatistics analysis on the microbiome data and the meta-analysis with human covariates. S.R., S.T., K.G., and M.W. wrote the article. Y.S., P.B., M.C., and W.A. revised and edited the draft. All authors discussed the results and commented on the article.

## Declaration of interests

The authors declare no competing interests.

## STAR★Methods

### Key resources table

**Table:** 

REAGENT or RESOURCE	SOURCE	IDENTIFIER
**Biological samples**		

Fecal samples	This study and Rampelli et al.^12^	N/A

**Critical commercial assays**		

DNeasy Blood & Tissue Kit	QIAGEN	Cat#69506
RNeasy PowerMicrobiome Kit	QIAGEN	Cat#26000
RNAClean XP Beads	Beckman Coulter	Cat#A66514
AMPure XP Beads	Beckman Coulter	Cat#A63881
NextSeq 500/550 High Output Kit v2.5 (300 Cycles)	Illumina	Cat#20024908
TruSeq Stranded Total RNA Library Kit	Illumina	Cat#RS-122-2201

**Deposited data**		

Human gut microbiome data	Rampelli et al.^12^	MGRAST (https://www.mg-rast.org/mgmain.html?mgpage=project&project=mgp84098)
Human gut microbiome data	This study	BioProject ID: PRJEB102780

**Software and algorithms**		

PANDAseq	Masella et al.^37^	https://github.com/neufeld/pandaseq
QIIME 2	Bolyen et al.^38^	https://qiime2.org/
DADA2	Callahan et al.^39^	https://github.com/benjjneb/dada2
VSEARCH	Rognes et al.^40^	https://github.com/torognes/vsearch
FlashWeave	Tackmann et al.^41^	https://github.com/meringlab/FlashWeave.jl
Cytoscpae	Shannon et al.^42^	https://github.com/cytoscape/cytoscape
Megahit (v1.2.9)	Li et al.^43^	https://github.com/voutcn/megahit
BASTA	Kahlke et al.^44^	https://github.com/timkahlke/BASTA
DIAMOND	Buchfink et al.^45^	https://github.com/bbuchfink/diamond
R v4.2.0	R software	http://www.r-project.org

### Experimental model and study participant details

#### Study approval

The present research was conducted in accordance with all institutional and governmental regulations regarding the ethical inclusion of enlisted volunteers. Approval was obtained from the relevant ethics committees in each of the centers where the fieldwork was conducted: Cyprus National Bioethics Committee, Cyprus, 12/Jul/2007, No. EEBK/EM/2007/16 and 21/Feb/ 2013, No. EEBK/ETI/2012/33; Tallinn Medical Research Ethics Committee (TMREC), Estonia, 14/Jun/2007, No. 1093 and 17/Jan/2013, No. 128; Ethic Commission of the University of Bremen, Germany, 16/Jan/2007 and 11/Dec/2012; Medical Research Council, Hungary, 21/Jun/2007, 22-156/2007-1018EKU and 18/ Dec/2012, 4536/2013/EKU; Regional Ethics Research Board in Gothenburg, Sweden, 30/Jul/2007, No. 264-07 and 10/Jan/2013, No. 927-12. Written informed consent was obtained from adolescents older than 12 years and their parents and/or legal guardians. Oral consent was obtained from younger children.

#### Study design and sample collection

The study included children and adolescents enrolled in the context of the follow-up study cohort ‘Identification and Prevention of Dietary- and Lifestyle-Induced Health Effects in Children and Infants’ (IDEFICS) and the project ‘Investigating the determinants of food choice, lifestyle and health in European children, adolescents and their parents’ (I.Family). The IDEFICS prospective cohort enrolled 16,229 children aged 2 to 9.9 years from kindergartens and schools in eight European countries (*i.e.*, Belgium, Cyprus, Estonia, Germany, Hungary, Italy, Spain, and Sweden). Specifically, the IDEFICS study included a baseline survey (T0, conducted between September 2007 and May 2008) and a follow-up survey two years later (T1, September 2009 - July 2010). The information collected in the surveys included dietary habits, physical activity, socio-demographic factors, clinical and physical examinations, and health outcomes. Subsequently, the I.Family follow-up project was established as an extension of the IDEFICS study, which was conducted four years later (T3, 2013 – 2014; T2 was not used as it only consisted of a questionnaire). The children enrolled at T0 and/or T1 were followed up at T3, and the survey was extended to their parents and siblings. For further details on the design and methods of these additional investigations, see the work of Ahrens and colleagues.^46^

In our study, a subgroup of 218 children/adolescents from the IDEFICS/I.Family cohorts was selected based on the availability of fecal samples and participation in at least one follow-up examination (Table 1). In particular, fecal samples were collected for the first time in 2010 from 218 individuals, corresponding to the second IDEFICS survey (T1) in five of the eight participating countries (*i.e.*, Cyprus, Estonia, Germany, Hungary, and Sweden). The second fecal sample collection took place four years later, during the second follow-up (T3), in the same countries that had previously joined the initiative, but only from 92 individuals (for a total of 310 fecal samples). Seventy individuals and their 140 fecal samples were analyzed in Rampelli et al.,^12^ while the remaining 148 individuals and their 170 fecal samples were analyzed in the present study. Importantly, all 218 participants underwent clinical assessments at both T1 and T3, including anthropometric measurements (weight and body mass index – BMI), biomarkers of low-grade inflammation, dietary habits, and lifestyle variables. Therefore, longitudinal clinical trajectories (e.g., change in weight status from T1 to T3) refer to the full cohort of 218 participants, whereas GM analyses were performed on the fecal samples available (218 at T1 and 92 at T3). Due to the small sample size at T3, the GM analyses were mainly cross-sectional, examining the links between GM configurations (alongside health, lifestyle behaviors, and diet) at baseline (T1) and subsequent weight status.

### Method details

#### Assessment of clinical data, physical activity, and dietary habits

Anthropometric data, blood pressure accelerometry, genetic data from saliva, and physiological blood markers were measured. Educational level and sleep duration (hours/night) were also considered. Physical activity was assessed using the Evenson MVPA score. Audio-visual media (AVM) usage time (*i.e.*, PC and TV) was calculated as hours per week and was used as a proxy for sedentary time, as it has been shown to be associated with accelerometry-derived sedentary time,^47^ and as a proxy for exposure to unhealthy food and diet marketing.^48^ Finally, a FFQ and a self-administered computer-assisted 24-h dietary recall were administered to obtain information on the daily frequency of consumption of each food category considered and macronutrient intake, respectively.^49^ Participants were stratified by measurement time and weight status as follows: (i) normal weight at T1 and T3, (ii) overweight/obese at T1 and T3, (iii) underweight at T1 and T3, (iv) normal weight at T1 and excess weight gain at T3, (v) downward change in weight category at T3 compared to T1, and (vi) underweight at T1 and normal weight at T3. We used the international (International Obesity Task Force; IOTF) BMI cut-offs. All those who have moved down at least one weight class were summarized in group (v).

#### Microbial DNA and RNA extraction

Microbial DNA was extracted from 250 mg of stool, as previously described.^50^ Briefly, each sample underwent three consecutive steps of: i) chemical lysis, by resuspension in 1 ml of lysis buffer (500 mM NaCl, 50 mM Tris-HCl pH 8, 50 mM EDTA, 4% [w/v] SDS); ii) mechanical lysis, by homogenization using a FastPrep instrument (MP Biomedicals) with three bead-beating steps at 5.5 movements sec^-1^ for 1 min after adding 0.5 g of 0.1-mm zirconia beads and four 3-mm glass beads (BioSpec Products) to the samples; and iii) thermal lysis, by incubation at 95°C for 15 min. Stool particles were pelleted by centrifugation at 13,000 rpm for 5 min, and nucleic acids were precipitated by sequential addition of 260 μl of 10 M ammonium acetate and one volume of isopropanol. Finally, the nucleic acid pellets were washed with 70% ethanol and resuspended in TE buffer. RNA was removed by treatment with 2 μl of DNase-free RNase (10 mg ml^-1^) at 37°C for 15 min. The subsequent steps of protein removal and DNA purification were performed using the DNeasy Blood & Tissue Kit (QIAGEN), following the manufacturer’s instructions. The quantity and quality of purified DNA were assessed using a NanoDrop ND-1000 spectrophotometer (NanoDrop Technologies).

RNA extraction was performed on a subset of samples (selected based on availability and representativeness of the three GM configurations) using the RNeasy PowerMicrobiome Kit (QIAGEN) according to the manufacturer's instructions. Briefly, 250 mg of stool was processed by addition of chemical lysis buffer (PM1/β-mercaptoethanol), followed by homogenization using a FastPrep instrument (MP Biomedicals) at 5.5 movements sec^-1^ for 1 min. DNA was removed by on-column DNase treatment, followed by a wash step to remove the enzyme and any digested nucleic acids. Purified RNA was eluted in RNase-free water. For each sample, rRNA was depleted using the Ribo-Zero Gold Kit for bacteria (Illumina) according to the manufacturer's instructions. Briefly, total RNA was hybridized using rRNA Removal Beads (Illumina), followed by clean-up using RNAClean XP Beads (Beckman Coulter) after the RNA denaturation step.

#### 16S rRNA amplicon sequencing and bioinformatic analysis

For library preparation, the V3-V4 hypervariable regions of the 16S rRNA gene were amplified using primers 341F and 785R, including overhang adapter sequences for Illumina sequencing, as previously reported by Rampelli et al.^12^ PCR products of approximately 460 bp were purified using a magnetic bead-based system (Agencourt AMPure XP; Beckman Coulter). Indexed libraries were prepared by limited-cycle PCR using Nextera technology, and further purified as described above. Final libraries were pooled at equimolar concentration, denatured with 0.2 N NaOH, and diluted to 5 pM with 20% PhiX control. Sequencing was performed on an Illumina MiSeq platform, using a 2 × 250 bp paired-end protocol, according to the manufacturer’s instructions (Illumina). To minimize batch effects due to different sequencing runs, all raw reads from the previous dataset^12^ and the newly generated dataset were processed together using the same bioinformatic pipeline. This combined PANDAseq^37^ and QIIME 2^38^ with DADA2^39^ for sequence filtering and VSEARCH^40^ for OTU clustering at 97% identity and taxonomic assignment. Alpha diversity was assessed using Faith’s phylogenetic diversity. For beta diversity, the weighted and unweighted UniFrac distance matrices were used as input for Principal Coordinates Analysis (PCoA). PCoA plots, heatmaps and bar plots were built using the R packages stat, made4 and vegan (http://www.cran.r-project.org/package=vegan). To control for potential batch effects, the sequencing run was included as a confounding factor in PERMANOVA models (adonis2, vegan package in R) on weighted and unweighted UniFrac distance matrices; the group effect (M1-M3) remined highly significant (P<0.0001), indicating a negligible batch effect. Networks were built using the genus-level abundance table from the QIIME 2 analysis and the FlashWeave software with default parameters.^41^ Cytoscape was used for network analysis and graphical representation.^42^ Connectivity values, specifically closeness centrality, betweenness centrality, and degree, for identifying keystone taxa from the microbial network were obtained from Cytoscape. Keystone taxa were those genera with the highest combination of closeness centrality, betweenness centrality and degree, with an average relative abundance >1%.

#### Shotgun metatranscriptomics and bioinformatic analysis

RNA libraries were prepared using the TruSeq Stranded Total RNA Library Kit (Illumina) according to the manufacturer's instructions. Briefly, after reverse transcription, synthesis of the second strand was performed using a combination of enzymes and buffer solutions to allow degradation of the RNA strand, generation of a second cDNA strand and generation of blunt DNA ends. Subsequent addition of the A-base ensured efficient ligation of Illumina-compatible adapters. The generated RNA-seq libraries were PCR amplified, purified using magnetic beads (Agencourt AMPure XP; Beckman Coulter) and pooled at an equimolar concentration of 4 nM before loading onto the flow cell. Sequencing was performed on an Illumina NextSeq 500 platform using a 2 × 150 bp paired-end protocol according to the manufacturer's instructions (Illumina). Metatranscriptomic reads were filtered following the procedures of the Human Microbiome Project.^51^ In particular, sequences derived from human contamination were filtered out using bmtagger (*Homo sapiens* reference NCBI GenBank accession: GCA_000001405.29). Reads were then processed using trimBWAstyle (TrimBWAstyle.usingBam.pl, 2010) for quality trimming (phred quality score <20) and length filtering (*i.e.*, reads <60 bp long were drop out). Finally, duplicate reads were removed using the Picard tool “EstimatedLibraryComplexity” (version 1.71). The resulting high-quality reads were assembled using megahit (version 1.2.9)^43^ with default parameters. Each contig was considered as a transcript with its own coverage corresponding to its abundance in the samples. BASTA^44^ was used to assign a taxonomy to each contig. Diamond^45^ was used to align the contigs to the functional database containing the most relevant functionalities of the previously identified keystone taxa (*i.e.*, *Faecalibacterium*, *[Ruminococcus]*, *Lachnospira* and *Bacteroides*) in terms of microbial effectors known to positively or negatively impact human health. The IDs of such genes are provided in supplemental information.

### Quantification and statistical analysis

Statistical analysis of the -omics datasets was performed using R Studio 1.2.1335 on R software version 4.2.0 (https://www.r-project.org/) and the packages “stats”, “vegan” (https://cran.r-project.org/package=vegan), and “quantreg” (https://cran.r-project.org/package=quantreg). GM configurations were identified through hierarchical Ward linkage clustering based on Spearman correlation coefficients of OTU proportions, filtered by prevalence of at least 30% of individuals. Multiple testing using the Benjamini-Hochberg method was performed to assess statistically significant within-group correlations associated with each cluster. The Spearman distance matrix was used as input for a permutational MANOVA test, performed using the “adonis” function of “vegan”, to test for statistically significant differences between the different clusters. Fisher’s exact test was used to assess the distribution of children/adolescents according to their weight status within the three GM clusters (M1 to M3), as well as within combinations of GM cluster and lifestyle behaviors (*i.e.*, diet and physical activity). For the latter analysis, individuals were stratified into high, medium or low HDS and MVPA groups, based on the corresponding values’ percentile distribution. Specifically, the high group comprised individuals whose HDS/MVPA values fell between the 75^th^ and 100^th^ percentile, the medium group comprised individuals whose HDS/MVPA values fell between the 26^th^ and 74^th^ percentile, and the low group comprised individuals whose HDS/MVPA values fell below the 25^th^ percentile. The Wilcoxon test was used to identify statistically significant differences in relative taxon abundance, alpha diversity, transcript abundance and dietary data between GM clusters. Statistically significant results (P < 0.05) were graphically indicated with an asterisk (∗). The permutation test with pseudo-*F* ratio (function “adonis” in “vegan”) was used to assess the significance of segregation of GM clusters in the PCoA. Correlations between GM composition and host metadata, including clinical measurements and behavioral parameters, in the unweighted UniFrac PCoA were analyzed using quantile (median) regression tests, adjusted for sex, age and country of origin. Missing data were handled using a complete-case approach separately for each analysis (no imputation was applied), meaning that only subjects missing a specific variable were excluded from analyses involving that variable. The “envfit” function of “vegan” was used to superimpose dietary data on the PCoA space to identify the food items that contributed most to the ordination space. P values were corrected for multiple testing using the Benjamini-Hochberg method, with a false discovery rate (FDR) ≤ 0.05 considered statistically significant.
