# Supplementary material for: Disrupted gut microbiome networks and unhealthy behaviors predict metabolic dysfunction in children and adolescents in the long term
Source: iScience. 2026 Jan 24;29(2):114763. doi: 10.1016/j.isci.2026.114763 (PMC12907672; doi:10.1016/j.isci.2026.114763)
Supplement: Document S1. Figure S1 and Tables S1–S3 [file mmc1.pdf]

iScience, Volume *29*

Supplemental information

Disrupted gut microbiome networks and unhealthy behaviors predict metabolic dysfunction in children and adolescents in the long term

Silvia Turroni, Kathrin Günther, Federica D'Amico, Toomas Veidebaum, Yiannis Kourides, Dénes Molnár, Lauren Lissner, Ronja Foraita, Monica Barone, Carlos Mora-Martínez, Yolanda Sanz, Arno Fraterman, Maike Wolters, Patrizia Brigidi, Marco Candela, Wolfgang Ahrens, and Simone Rampelli

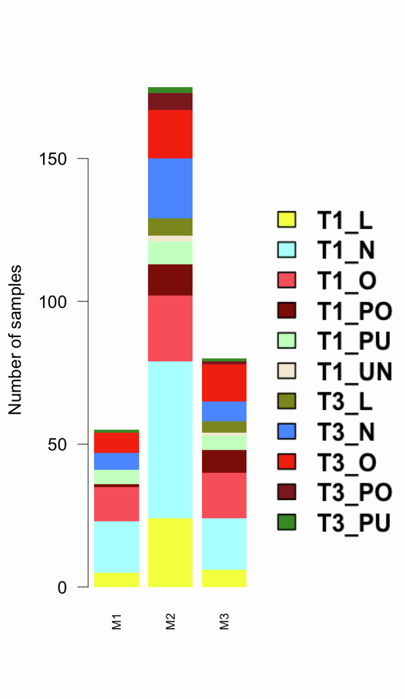

Supplementary Figure 1. Gut microbiome configurations in children/adolescents in relation to time and weight status. Bar plots showing the number of children/adolescents included in each gut microbiome configuration (M1 to M3), stratified by time and weight status. Color coding: yellow, T1 individuals who showed a downward change in weight status at T3 compared to T1 (T1_L); cyan, individuals with normal weight at T1 who were also normal weight at T3 (T1_N); light red, individuals with normal weight at T1 who developed obesity at T3 (T1_O); dark red, individuals with obesity at T1 who were also obese at T3 (T1_PO); light green, individuals with underweight at T1 who were also underweight at T3 (T1_PU); beige, individuals with underweight at T1 who returned to normal weight at T3 (T1_UN); army green, T3 individuals who showed a downward change in weight status at T3 compared to T1 (T3_L); light blue, individuals with normal weight at T3 who were also normal weight at T1 (T3_N); red, individuals with obesity at T3 who were normal weight at T1 (T3_O); heavy dark red, individuals with obesity at T3 who were also obese at T1 (T3_PO); and forest green, individuals with underweight at T3 who were also underweight at T1 (T3_PU). See also Supplementary Table 3.

Supplementary Table 1. Characteristics of children/adolescents with stool samples at T1 and T3. Participants were stratified by measurement time and weight status as follows: i) normal weight at T1 and excess weight gain at T3; ii) normal weight at T1 and T3; iii) overweight/obese at T1 and T3; iv) underweight at T1 and T3; and v) downward change in weight category at T3 compared to T1.

	Normal weight at T1 and excess weight gain at T3		Normal weight at T1 and T3		Overweight/obese at T1 and T3		Underweight at T1 and T3		Downward change in weight category		All	
	N	% (row)	N	% (row)	N	% (row)	N	% (row)	N	% (row)	N	% (row)
Total	38	41.30	33	35.87	7	7.61	4	4.35	10	10.87	92	100.00
	N	% (column)	N	% (column)	N	% (column)	N	% (column)	N	% (column)	N	% (column)
Sex of the child (male)	16	42.11	19	57.58	4	57.14	4	100.00	7	70.00	50	54.35
	N	Mean	N	Mean	N	Mean	N	Mean	N	Mean	N	Mean
Age (years) - T1	38	7.83	33	7.08	7	9.43	4	6.8	10	8.55	92	7.72
Age (years) - T3	38	11.69	33	11.10	7	13.3	4	10.8	10	12.6	92	11.66
Body Mass Index (kg/m^2) - T1	38	15.82	33	16.03	7	24.33	4	13.58	10	20.01	92	16.9
Body Mass Index (kg/m^2) - T3	38	19.95	33	17.38	7	27.16	4	14.48	10	20.5	92	19.4
Weight of participant (kg) - T1	38	27.93	33	26.35	7	52.29	4	20.45	10	39.28	92	30.12
Weight of participant (kg) - T3	38	48.65	33	40.90	7	76.37	4	31.3	10	54.77	92	47.89
Height of participant (cm) - T1	38	131.19	33	127.35	7	144.99	4	122.03	10	137.62	92	131.16
Height of participant (cm) - T3	38	154.44	33	152.42	7	166.63	4	146.08	10	161.61	92	155.06

Supplementary Table 2. Characteristics of children/adolescents with stool samples only at T1. Participants were stratified by measurement time and weight status as follows: i) normal weight at T1 and excess weight gain at T3; ii) normal weight at T1 and T3; iii) overweight/obese at T1 and T3; iv) underweight at T1 and T3; v) underweight at T1 and normal weight at T3; and vi) downward change in weight category at T3 compared to T1.

	Normal weight at T1 and excess weight gain at T3		Normal weight at T1 and T3		Overweight/obese at T1 and T3		Underweight at T1 and T3		Underweight at T1 and normal weight at T3		Downward change in weight category		All	
	N	% (row)	N	% (row)	N	% (row)	N	% (row)	N	% (row)	N	% (row)	N	% (row)
Total	17	13.49	56	44.44	12	9.52	14	11.11	3	2.38	24	19.05	126	100.00
	N	% (column)	N	% (column)	N	% (column)	N	% (column)	N	% (column)	N	% (column)	N	% (column)
Sex of the child (male)	9	52.94	33	58.93	4	33.33	8	57.14	2	66.67	11	45.83	55	53.17
	N	Mean	N	Mean	N	Mean	N	Mean	N	Mean	N	Mean	N	Mean
Age (years) - T1	17	8.72	56	8.43	12	7.73	14	8.04	3	8.03	24	8.62	126	8.39
Age (years) - T3	17	12.56	56	12.15	12	11.36	14	11.72	3	11.57	24	12.48	126	12.13
Body Mass Index (kg/m^2) - T1	17	15.99	56	16.43	12	21.15	14	13.6	3	14.1	24	17.59	126	16.67
Body Mass Index (kg/m^2) - T3	17	20.07	56	17.98	12	24.99	14	14.89	3	16.37	24	18.01	126	18.55
Weight of participant (kg) - T1	17	28.11	56	29.53	12	39.11	14	22.87	3	23.7	24	33.29	126	30.09
Weight of participant (kg) - T3	17	48.61	56	44.47	12	62.7	14	33.54	3	39.2	24	46.36	126	45.78
Height of participant (cm) - T1	17	132.25	56	133.35	12	134.86	14	129.44	3	129.2	24	135.59	126	133.24
Height of participant (cm) - T3	17	155.15	56	156.18	12	157.25	14	149.57	3	152.7	24	158.6	126	155.78

Supplementary Table 3. Summary of the recruited children/adolescents, along with information on gender, age, weight group and gut microbiome cluster of their fecal samples. Those without an "m" before their ID were from our previous publication [S1]. Weight group: T1 individuals who showed a downward change in weight status at T3 compared to T1 (T1_L); individuals with normal weight at T1 who were also normal weight at T3 (T1_N); individuals with normal weight at T1 who developed obesity at T3 (T1_O); individuals with obesity at T1 who were also obese at T3 (T1_PO); individuals with underweight at T1 who were also underweight at T3 (T1_PU); individuals with underweight at T1 who returned to normal weight at T3 (T1_UN); T3 individuals who showed a downward change in weight status at T3 compared to T1 (T3_L); individuals with normal weight at T3 who were also normal weight at T1 (T3_N); individuals with obesity at T3 who were normal weight at T1 (T3_O); individuals with obesity at T3 who were also obese at T1 (T3_PO); and individuals with underweight at T3 who were also underweight at T1 (T3_PU). Gut microbiome cluster: M1 to M3. The previous assignment to the C1–C4 clusters is provided for samples from Rampelli et al. [S1]. See also Supplementary Figure 1.

Sample ID	Gender	Age (years)	Individual ID	Weight group	Gut microbiome cluster from the present study	Gut microbiome cluster from Rampelli et al. [S1]
IDE13	Male	9.4	2	T1_N	M2	C1
IFA54	Male	13.1	2	T3_N	M2	C2
IDE18	Female	9.4	3	T1_N	M2	C2
IFA62	Female	13.6	3	T3_N	M2	C2
IDE10	Female	6.5	6	T1_O	M2	C2
IFA45	Female	10.7	6	T3_O	M2	C2
IDE17	Male	9.3	7	T1_O	M2	C1
IFA50	Male	13.3	7	T3_O	M2	C1
IDE19	Female	9.1	9	T1_O	M2	C2
IFA46	Female	13	9	T3_O	M2	C2
IDE1	Male	10.2	11	T1_N	M2	C2
IFA59	Male	14.6	11	T3_N	M2	C2
IDE3	Female	9.3	12	T1_O	M3	C3
IFA47	Female	13.3	12	T3_O	M3	C1
IDE16	Female	9.7	14	T1_N	M2	C2
IFA63	Female	13.9	14	T3_N	M3	C2
IDE15	Female	9.5	16	T1_N	M2	C1
IFA60	Female	13.7	16	T3_N	M2	C1
IDE7	Female	10	17	T1_O	M3	C2
IFA65	Female	13.6	17	T3_O	M3	C3
IDE14	Female	9.5	18	T1_O	M2	C1
IFA44	Female	13.8	18	T3_O	M3	C1
IDE61	Male	10	20	T1_N	M2	C2
IFA52	Male	13.8	20	T3_N	M2	C2
IDE62	Male	6.7	21	T1_N	M2	C1
IFA68	Male	10.8	21	T3_N	M2	C2

IDE64	Female	9.2	22	T1_O	M2	C1
IFA67	Female	13	22	T3_O	M3	C1
IDE68	Female	9.9	23	T1_O	M2	C1
IFA51	Female	13.7	23	T3_O	M2	C1
IDE59	Male	9.7	24	T1_N	M1	C3
IFA56	Male	13.5	24	T3_N	M1	C3
IDE48	Male	10.2	26	T1_N	M3	C1
IFA49	Male	14.1	26	T3_N	M2	C2
IDE60	Female	9.7	27	T1_O	M2	C2
IFA53	Female	13.5	27	T3_O	M2	C2
IDE65	Female	4.6	29	T1_N	M2	C1
IFA57	Female	8.5	29	T3_N	M2	C2
IDE9	Male	8	30	T1_N	M2	C1
IFA58	Male	12	30	T3_N	M2	C1
IDE63	Female	9.4	31	T1_O	M2	C2
IFA55	Female	13.2	31	T3_O	M2	C2
IDE70	Male	4.6	33	T1_N	M2	C2
IFA61	Male	8.6	33	T3_N	M2	C2
IDE4	Male	6	34	T1_N	M2	C3
IFA13	Male	10.2	34	T3_N	M2	C2
IDE50	Female	6.4	35	T1_N	M2	C2
IFA25	Female	10.5	35	T3_N	M2	C2
IDE56	Male	6.8	36	T1_N	M1	C3
IFA30	Male	10.8	36	T3_N	M3	C1
IDE55	Female	6.7	37	T1_O	M2	C2
IFA64	Female	10.7	37	T3_O	M2	C1
IDE52	Female	5.8	39	T1_O	M3	C1
IFA17	Female	9.8	39	T3_O	M2	C1
IDE49	Female	7	40	T1_N	M2	C1
IFA9	Female	11.1	40	T3_N	M2	C2
IDE6	Female	9.1	41	T1_N	M1	C2
IFA10	Female	13.4	41	T3_N	M1	C3
IDE53	Male	5.8	46	T1_N	M2	C2
IFA24	Male	9.7	46	T3_N	M2	C1
IDE51	Female	4.7	48	T1_N	M2	C2
IFA28	Female	8.9	48	T3_N	M2	C1
IDE2	Male	4.7	49	T1_N	M2	C3
IFA33	Male	8.9	49	T3_N	M2	C1
IDE66	Female	6.8	51	T1_O	M2	C2
IFA48	Female	11	51	T3_O	M2	C1
IDE67	Male	4.8	52	T1_N	M2	C1
IFA23	Male	8.9	52	T3_N	M2	C1
IDE5	Female	5.5	53	T1_N	M2	C2
IFA18	Female	9.5	53	T3_N	M2	C2
IDE11	Female	6.9	54	T1_N	M2	C2
IFA20	Female	10.8	54	T3_N	M2	C2
IDE57	Female	5.8	55	T1_N	M2	C2

IFA26	Female	9.8	55	T3_N	M2	C2
IDE58	Male	6.1	57	T1_N	M2	C1
IFA29	Male	10.3	57	T3_N	M2	C2
IDE12	Female	4.9	58	T1_O	M2	C3
IFA21	Female	8.9	58	T3_O	M2	C1
IDE8	Male	5.1	59	T1_N	M2	C1
IFA22	Male	8.9	59	T3_N	M3	C1
IDE69	Female	5.1	60	T1_O	M2	C2
IFA66	Female	9	60	T3_O	M3	C1
IDE54	Male	5.2	61	T1_O	M2	C2
IFA19	Male	9.1	61	T3_O	M1	C3
IDE25	Male	8.8	62	T1_N	M1	C4
IFA12	Male	12.7	62	T3_N	M1	C4
IDE28	Female	4.3	63	T1_N	M1	C4
IFA16	Female	8.2	63	T3_N	M1	C4
IDE32	Male	8.7	65	T1_O	M3	C3
IFA32	Male	12.9	65	T3_O	M3	C3
IDE27	Male	9.5	66	T1_N	M3	C1
IFA31	Male	13.8	66	T3_N	M3	C1
IDE26	Male	7.9	67	T1_O	M3	C3
IFA11	Male	11.7	67	T3_O	M3	C3
IDE42	Male	4.7	69	T1_O	M3	C3
IFA27	Male	8.5	69	T3_O	M3	C3
IDE20	Female	9.6	71	T1_O	M2	C3
IFA6	Female	13.7	71	T3_O	M2	C1
IDE33	Female	5.8	72	T1_N	M3	C3
IFA3	Female	9.7	72	T3_N	M3	C1
IDE21	Male	8.5	73	T1_O	M2	C2
IFA1	Male	12.2	73	T3_O	M2	C3
IDE38	Female	8.6	74	T1_O	M2	C3
IFA2	Female	12.4	74	T3_O	M2	C2
IDE40	Male	5.6	75	T1_N	M1	C3
IFA4	Male	9.3	75	T3_N	M1	C3
IDE39	Male	6.1	76	T1_O	M1	C4
IFA14	Male	9.4	76	T3_O	M1	C3
IDE31	Male	4.7	77	T1_N	M1	C4
IFA5	Male	8.8	77	T3_N	M3	C1
IDE29	Male	10.3	78	T1_O	M2	C2
IFA43	Male	14.1	78	T3_O	M2	C2
IDE30	Male	5.9	80	T1_O	M3	C3
IFA69	Male	9.7	80	T3_O	M1	C4
IDE37	Female	6.2	81	T1_O	M1	C4
IFA42	Female	10	81	T3_O	M1	C3
IDE24	Male	11.1	82	T1_O	M3	C3
IFA70	Male	15.2	82	T3_O	M1	C4
IDE22	Male	11	83	T1_O	M1	C3
IFA35	Male	15	83	T3_O	M1	C3

IDE41	Female	9.8	84	T1_N	M1	C4
IFA8	Female	13.8	84	T3_N	M1	C4
IDE36	Male	6.6	85	T1_O	M3	C1
IFA37	Male	10.5	85	T3_O	M2	C2
IDE35	Male	9.3	86	T1_O	M3	C3
IFA41	Male	13.4	86	T3_O	M3	C3
IDE43	Female	9.9	87	T1_O	M3	C3
IFA38	Female	13.2	87	T3_O	M3	C3
IDE45	Male	7.2	88	T1_O	M1	C4
IFA39	Male	10.5	88	T3_O	M3	C3
IDE46	Male	8	91	T1_O	M1	C4
IFA7	Male	11.8	91	T3_O	M1	C4
IDE23	Male	4.4	93	T1_N	M2	C1
IFA15	Male	7.9	93	T3_N	M3	C3
IDE44	Female	5.5	95	T1_O	M1	C3
IFA36	Female	8.8	95	T3_O	M3	C3
IDE47	Female	5.5	96	T1_O	M1	C4
IFA34	Female	8.8	96	T3_O	M2	C4
IDE34	Female	7.4	97	T1_O	M1	C3
IFA40	Female	8.9	97	T3_O	M3	C3
IDE145	Female	10	m1	T1_PO	M2	New sample
IFA71	Female	14	m1	T3_PO	M2	New sample
IDE154	Female	10.1	m10	T1_PO	M2	New sample
IDE168	Female	10.5	m100	T1_N	M1	New sample
IDE169	Male	9.1	m101	T1_N	M3	New sample
IDE170	Female	7.4	m102	T1_PU	M2	New sample
IDE171	Male	9.4	m103	T1_O	M3	New sample
IDE172	Female	9.6	m104	T1_O	M3	New sample
IDE173	Male	10.3	m105	T1_N	M2	New sample
IDE174	Male	10.2	m106	T1_UN	M3	New sample
IDE175	Male	10.3	m107	T1_N	M3	New sample
IDE176	Female	7.5	m108	T1_PO	M2	New sample
IDE177	Male	7.3	m109	T1_N	M2	New sample
IDE161	Female	9.2	m11	T1_L	M3	New sample
IDE178	Male	8.6	m110	T1_O	M2	New sample
IDE180	Female	8.7	m112	T1_N	M2	New sample
IDE181	Female	8.6	m113	T1_N	M2	New sample
IDE182	Female	8.8	m114	T1_N	M2	New sample
IDE183	Male	10.1	m115	T1_N	M2	New sample
IDE184	Female	9	m116	T1_N	M1	New sample
IDE185	Female	8.6	m117	T1_O	M3	New sample
IDE186	Male	9.2	m118	T1_N	M3	New sample
IDE187	Female	8.6	m119	T1_O	M2	New sample
IDE150	Male	9.8	m12	T1_PO	M3	New sample
IFA82	Male	13.6	m12	T3_PO	M2	New sample
IDE188	Male	5.7	m120	T1_N	M1	New sample
IDE189	Male	7.6	m121	T1_O	M3	New sample

IDE190	Male	8.5	m122	T1_L	M2	New sample
IDE191	Male	7.2	m123	T1_N	M2	New sample
IDE192	Male	9.8	m124	T1_L	M3	New sample
IDE193	Male	9.3	m125	T1_O	M2	New sample
IDE194	Male	6.6	m126	T1_N	M1	New sample
IDE195	Female	5.5	m127	T1_PU	M1	New sample
IDE196	Female	8.8	m128	T1_N	M1	New sample
IDE197	Female	9	m129	T1_PU	M2	New sample
IDE152	Male	10.8	m13	T1_PO	M3	New sample
IFA83	Male	14.6	m13	T3_PO	M2	New sample
IDE199	Male	8.8	m130	T1_N	M3	New sample
IDE202	Male	8.3	m131	T1_PO	M3	New sample
IDE203	Female	4.9	m132	T1_L	M1	New sample
IDE205	Male	10.6	m133	T1_N	M2	New sample
IDE207	Male	7.3	m134	T1_PU	M1	New sample
IDE208	Male	9.5	m135	T1_PU	M3	New sample
IDE209	Male	6.9	m136	T1_N	M1	New sample
IDE210	Female	6.6	m137	T1_PU	M1	New sample
IDE211	Female	6.9	m138	T1_L	M1	New sample
IDE212	Female	7.7	m139	T1_L	M3	New sample
IDE156	Female	10.3	m14	T1_L	M2	New sample
IFA84	Female	14	m14	T3_L	M2	New sample
IDE213	Female	10.2	m140	T1_PO	M2	New sample
IDE214	Female	9.1	m141	T1_L	M2	New sample
IDE215	Female	9.9	m142	T1_N	M3	New sample
IDE216	Male	9.6	m143	T1_N	M2	New sample
IDE217	Female	9.8	m144	T1_L	M2	New sample
IDE218	Female	6.1	m145	T1_N	M2	New sample
IDE219	Female	6	m146	T1_N	M2	New sample
IDE220	Female	9.9	m147	T1_PO	M3	New sample
IDE200	Female	6.7	m148	T1_PO	M2	New sample
IDE201	Male	8	m149	T1_PU	M1	New sample
IDE132	Male	10.1	m15	T1_L	M2	New sample
IFA85	Male	13.9	m15	T3_L	M3	New sample
IDE204	Male	7.6	m150	T1_PO	M3	New sample
IDE143	Male	4.8	m16	T1_PO	M2	New sample
IFA86	Male	8.6	m16	T3_PO	M2	New sample
IDE158	Male	9.3	m17	T1_PU	M3	New sample
IFA87	Male	13.5	m17	T3_PU	M1	New sample
IDE147	Male	11	m18	T1_PO	M2	New sample
IFA88	Male	14.7	m18	T3_PO	M2	New sample
IDE136	Male	4.4	m19	T1_L	M2	New sample
IFA89	Male	8.6	m19	T3_L	M2	New sample
IDE162	Male	5.2	m2	T1_PU	M2	New sample
IFA72	Male	9.5	m2	T3_PU	M3	New sample
IDE139	Male	6.9	m20	T1_L	M1	New sample
IFA90	Male	11.1	m20	T3_L	M3	New sample

IDE159	Male	10.1	m21	T1_L	M2	New sample
IFA91	Male	14.4	m21	T3_L	M2	New sample
IDE146	Male	9.3	m22	T1_L	M2	New sample
IFA92	Male	13.6	m22	T3_L	M2	New sample
IDE130	Male	6.1	m23	T1_PU	M2	New sample
IFA93	Male	10.1	m23	T3_PU	M2	New sample
IDE95	Female	9.4	m24	T1_L	M1	New sample
IFA94	Female	13.6	m24	T3_L	M3	New sample
IDE71	Male	9.3	m25	T1_N	M3	New sample
IDE72	Female	7.5	m26	T1_PO	M3	New sample
IDE73	Female	8.9	m27	T1_N	M3	New sample
IDE74	Female	8.7	m28	T1_N	M2	New sample
IDE75	Female	8.7	m29	T1_N	M2	New sample
IDE138	Female	6	m3	T1_L	M2	New sample
IFA73	Female	10	m3	T3_L	M2	New sample
IDE76	Male	9.5	m30	T1_PU	M3	New sample
IDE77	Male	9.3	m31	T1_N	M3	New sample
IDE79	Male	9.9	m33	T1_L	M2	New sample
IDE80	Male	8.3	m34	T1_N	M2	New sample
IDE81	Female	9.9	m35	T1_N	M2	New sample
IDE82	Male	7.4	m36	T1_L	M2	New sample
IDE83	Female	8.4	m37	T1_N	M2	New sample
IDE84	Female	9.1	m38	T1_UN	M2	New sample
IDE85	Female	5.3	m39	T1_PO	M2	New sample
IDE155	Female	9.8	m4	T1_O	M2	New sample
IFA74	Female	14.1	m4	T3_O	M2	New sample
IDE86	Female	9	m40	T1_O	M1	New sample
IDE87	Female	7.6	m41	T1_L	M2	New sample
IDE88	Female	5.3	m42	T1_N	M2	New sample
IDE89	Male	9.2	m43	T1_N	M2	New sample
IDE90	Female	10.5	m44	T1_L	M2	New sample
IDE91	Male	7	m45	T1_O	M1	New sample
IDE92	Male	6.8	m46	T1_PU	M3	New sample
IDE93	Female	8.4	m47	T1_L	M2	New sample
IDE94	Male	8.3	m48	T1_N	M2	New sample
IDE97	Female	4.4	m49	T1_O	M1	New sample
IDE198	Male	8.4	m5	T1_L	M1	New sample
IFA75	Male	12.4	m5	T3_L	M3	New sample
IDE98	Male	6.2	m50	T1_PO	M3	New sample
IDE99	Male	9.7	m51	T1_N	M3	New sample
IDE100	Female	10	m52	T1_O	M3	New sample
IDE101	Male	6.4	m53	T1_PU	M3	New sample
IDE102	Female	9.4	m54	T1_PO	M2	New sample
IDE103	Female	7.2	m55	T1_L	M2	New sample
IDE104	Female	9.1	m56	T1_N	M3	New sample
IDE105	Male	6.2	m57	T1_N	M2	New sample
IDE106	Female	9.3	m58	T1_PU	M2	New sample

IDE107	Male	8.7	m59	T1_PO	M3	New sample
IDE96	Male	6.6	m6	T1_PU	M2	New sample
IFA76	Male	10.1	m6	T3_PU	M2	New sample
IDE108	Male	9.7	m60	T1_N	M1	New sample
IDE109	Male	4.8	m61	T1_UN	M2	New sample
IDE110	Male	7.6	m62	T1_PU	M1	New sample
IDE111	Male	10.2	m63	T1_L	M2	New sample
IDE112	Male	7.8	m64	T1_N	M2	New sample
IDE113	Male	7.6	m65	T1_N	M3	New sample
IDE114	Female	7.1	m66	T1_N	M3	New sample
IDE115	Female	9.7	m67	T1_PU	M2	New sample
IDE116	Female	8.7	m68	T1_N	M2	New sample
IDE117	Male	9.2	m69	T1_N	M2	New sample
IDE206	Male	10.6	m7	T1_L	M2	New sample
IFA77	Male	14.4	m7	T3_L	M2	New sample
IDE118	Male	10.4	m70	T1_O	M1	New sample
IDE119	Male	8.7	m71	T1_L	M3	New sample
IDE120	Female	5.7	m72	T1_N	M3	New sample
IDE121	Female	9.8	m73	T1_O	M2	New sample
IDE122	Female	8.8	m74	T1_N	M2	New sample
IDE123	Female	9.1	m75	T1_L	M2	New sample
IDE124	Female	7.7	m76	T1_N	M2	New sample
IDE125	Female	10.5	m77	T1_L	M2	New sample
IDE126	Male	4.3	m78	T1_L	M2	New sample
IDE127	Female	8.2	m79	T1_N	M1	New sample
IDE133	Female	9.9	m8	T1_PO	M2	New sample
IFA78	Female	13.9	m8	T3_PO	M2	New sample
IDE128	Male	8.8	m80	T1_O	M2	New sample
IDE129	Male	10.7	m81	T1_L	M2	New sample
IDE131	Male	9.9	m82	T1_L	M2	New sample
IDE134	Male	4.5	m83	T1_N	M2	New sample
IDE135	Male	5.5	m84	T1_N	M2	New sample
IDE140	Female	10	m85	T1_L	M2	New sample
IDE141	Male	10.3	m86	T1_L	M3	New sample
IDE142	Female	5.5	m87	T1_PO	M1	New sample
IDE144	Male	9.5	m88	T1_N	M2	New sample
IDE148	Male	9.6	m89	T1_N	M2	New sample
IDE137	Female	9.7	m9	T1_PO	M2	New sample
IFA79	Female	13.7	m9	T3_PO	M3	New sample
IDE149	Male	9.9	m90	T1_PU	M2	New sample
IDE151	Male	4.1	m91	T1_N	M2	New sample
IDE153	Female	9.8	m92	T1_N	M1	New sample
IDE157	Male	10.3	m93	T1_N	M2	New sample
IDE160	Male	5.1	m94	T1_L	M2	New sample
IDE163	Male	10.1	m95	T1_N	M3	New sample
IDE164	Male	9.4	m96	T1_N	M1	New sample
IDE165	Female	10.3	m97	T1_L	M3	New sample

IDE166	Male	9.8	m98	T1_N	M3	New sample
IDE167	Male	8.4	m99	T1_N	M2	New sample

References

[S1] Rampelli, S., Guenther, K., Turroni, S., Wolters, M., Veidebaum, T., Kourides, Y., Molnár, D., Lissner, L., Benitez-Paez, A., Sanz, Y., et al. (2018). Pre-obese children's dysbiotic gut microbiome and unhealthy diets may predict the development of obesity. Commun. Biol. *1*, 222. https://doi.org/10.1038/s42003-018-0221-5.
